# Reliable Screening of Dye Phototoxicity by Using a *Caenorhabditis elegans* Fast Bioassay

**DOI:** 10.1371/journal.pone.0128898

**Published:** 2015-06-03

**Authors:** Javier Ignacio Bianchi, Juan Carlos Stockert, Lucila Ines Buzz, Alfonso Blázquez-Castro, Sergio Hernán Simonetta

**Affiliations:** 1 Fundación Instituto Leloir, CONICET. Patricias Argentinas 435, Buenos Aires, Argentina; 2 Departamento de Biología, Facultad de Ciencias, Universidad Autónoma de Madrid, Cantoblanco, Madrid, España; 3 Aarhus Institute of Advanced Studies (AIAS), Aarhus University, Aarhus, Denmark; CSIR-Central Drug Research Institute, INDIA

## Abstract

Phototoxicity consists in the capability of certain innocuous molecules to become toxic when subjected to suitable illumination. In order to discover new photoactive drugs or characterize phototoxic pollutants, it would be advantageous to use simple biological tests of phototoxicy. In this work, we present a pilot screening of 37 dyes to test for phototoxic effects in the roundworm *Caenorhabditis elegans*. Populations of this nematode were treated with different dyes, and subsequently exposed to 30 min of white light. Behavioral outcomes were quantified by recording the global motility using an infrared tracking device (WMicrotracker). Of the tested compounds, 17 dyes were classified as photoactive, being phloxine B, primuline, eosin Y, acridine orange and rose Bengal the most phototoxic. To assess photoactivity after uptake, compounds were retested after washing them out of the medium before light irradiation. Dye uptake into the worms was also analyzed by staining or fluorescence. All the positive drugs were incorporated by animals and produced phototoxic effects after washing. We also tested the stress response being triggered by the treatments through reporter strains. Endoplasmic reticulum stress response (hsp-4::GFP strain) was activated by 22% of phototoxic dyes, and mitochondrial stress response (hsp-6::GFP strain) was induced by 16% of phototoxic dyes. These results point to a phototoxic perturbation of the protein functionality and an oxidative stress similar to that reported in cell cultures. Our work shows for the first time the feasibility of *C*. *elegans* for running phototoxic screenings and underscores its application on photoactive drugs and environmental pollutants assessment.

## Introduction

The phototoxic properties of many compounds were demonstrated long ago [1], consisting in the capability of certain molecules to be activated when subjected to suitable illumination. When this effect requires molecular oxygen it has been named photodynamic action, and allowed the development of a new cancer treatment called “photodynamic therapy” (“PDT”) [2]. In a PDT treatment patients are treated with the drug and, once absorbed, the tumor area is subjected to light irradiation in order to activate drug phototoxicity and destroy cancer cells. The molecular mechanism of photoactivation is based on the generation of reactive oxygen species (ROS, and mainly singlet oxygen, ^1^O_2_), which damage cellular structures and induce cell death, either by apoptosis or necrosis [3–5]. Specific photoactivable drugs have been discovered and are currently used in human healthcare [6], and several common dyes have shown photodynamic activity. Some well characterized molecular structures with photoactive properties are thiazine [7–9], xanthene [10], acridine [11, 12], and triarylmethane [13] derivatives.

At the moment, most of the work on photoactivity of dyes has been concerned with biomedical applications [14], particularly PDT of cancer [4, 15, 16]. However, this particular effect spreads to other fields of interest such as photosterilization of water and blood products [9, 12], and environmental pollution [17–20]. It is worth to note that dyes used in industrial activities often present mutagenic, carcinogenic, and genotoxic photoactivity [15, 21, 22], which can result in a relevant risk for both human and environmental health.

In addition, xenobiotics such as herbicides, pesticides, aromatic hydrocarbons, cosmetics and personal care products are also photoactive environmental pollutants that, when illuminated, exert adverse effects on the quality of river and lake water, soil sediments and living organisms [23–29].

Biological models, from microorganisms to higher organisms, have been thoroughly employed to evaluate phototoxic effects of chemicals in order to explore the potential of new drugs for PDT or to prevent environmental damage from xenobiotics. Several bioassays for the detection of photodynamic effects are currently on use, examples being *Paramecium* [30], *Candida* [31], *Allium* [32], *Drosophila* [22], *Daphnia*, sea urchin [10], amphibian embryos [33], cell cultures [34], erythrocyte hemolysis [35], and intradermal tests [36].

In order to assess more precisely the phototoxicity of compounds such as drugs or pollutants, the use of a small translucent whole organism seems to be advantageous for simple, rapid and cheap bioassays. One of the simplest animal models is the nematode *Caenorhabditis elegans*, which has a fully sequenced genome, rudimentary organs similar to those found in mammals, and highly conserved cellular signaling pathways [37]. Also, the ease to culture, growth and reproduction of this organism has helped to establish it as a very suitable biological model for a variety of assessments. The main attraction of *C*. *elegans* for use in pharmacological and toxicological screening consists in the capability to be cultured in microplates (96 or 384 well plates), with small amounts of liquid medium, where animals can be incubated with experimental compounds [38].

In a whole animal approach, treatment with toxic doses will cause death, whereas subtoxic effects will affect animal behavior. Based on this premise, we employed here a motility tracking system, previously developed in our laboratory [39], to characterize the effects of thirty seven dyes, some already known to have phototoxic properties. As far as we know, this is the first time that the *C*. *elegans* model has been used for screening of this kind of compound.

## Materials and Methods

### Animal culture and strains

For the phototoxicity, uptake assays, and for qRT-PCR experiments, synchronized populations of *Caenorhabditis elegans* SS104 strain (glp-4 [bn2], a temperature sensitive sterile mutant) were cultured at non permissive temperature (25°C) in Nematode Growth Medium as described previously [40]. On the 1^st^ day of adult stage, animals were washed twice in saline solution (modified K-medium: 52 mM NaCl and 32 mM KCl [41] + 0.01% Triton X-100) and transferred to flat bottomed 96-well microplates (Greiner Bio-one Cat. 655101). An average of 50 animals was used per well with a final volume of 100 μl.

For the stress response assays the reporter lines used were zcIs4 [hsp-4::GFP], zcIs9 [hsp-60::GFP], zcIs13 [hsp-6::GFP] (SJ4005, SJ4100 and SJ4058 strains, respectively). For worm cultures the same protocol was followed but at 20°C. All strains were obtained from the *Caenorhabditis* Genetic Center, University of Minnesota, USA.

### Behavioral phototoxicity measurement

One hour after pipetting, basal motility of worms within the wells was assessed using an infrared tracking device (WMicrotracker, Designplus SRL, Argentina) [39]. This basal activity was recorded to normalize any subsequent activity variations to that initial activity, eliminating differences between wells due to population size. After basal measurement, dyes were added to the culture medium at final concentrations of 0.1, 1, 10 or 100 μM and incubated for 1 h before light irradiation (where applied). Dye photoactivation was carried out exposing the microplates for 30 min under a fluorescent white light source (2700°K white fluorescent lamp R7s 20W, Sica, Argentina) at 10 mW/cm^2^ of intensity. An additional water IR-filter (3-cm wide) was used to avoid heating, as previously reported [20].

Locomotor activity was tracked continuously in darkness during the incubation with the dye and for 4 h after dye activation. At least 4 replicate wells were used for each experiment, and the reported concentration was repeated independently three times, unless otherwise mentioned in the text.

### Chemical treatment

The dyes employed in this study, as well as their characteristics and known properties, are shown in Table 1. After a preliminary determination, compounds were used at 100 μM concentration (unless otherwise indicated).

**Table 1:** List of dyes used in the present work, with reference to the chemical group, Colour Index number (C.I.), electric charge (Z), source, and previously reported photoactivity (if any, and only for well known cases).

Dye (abbreviation)	Chemical group	C.I.	Z	Source	Photo Activity	Reference ^a^
1. Acridine orange (AO)	Acridine	46005	+	Merck	+	Zdolsek *et al*. 1990, Herkovits *et al*. 2007, Alvarez *el al*. 2011
2. Alizarin red S (ARS)	Anthraquinone	58005	−	Fluka	−	Seliger & McElroy 1965, Redmond & Gamlin 1999
3. Auramine O (AuO)	Diphenylmethane	41000	+	Analema	+	Stockert *et al*. 1990
4. Berberine (Ber)	Isoquinoline	75169	+	Sigma	+	Molero *et al*. 1985, Inbaraj *et al*. 2001
5. Bismarck brown Y (BBY)	Bis-azo	21000	2+	R.A. Lamb	−	Barbosa & Peters 1971
6. Carmine (Car)	Anthraquinone	75470	0	Gurr	nd	
7. Chrysoidine (Chr)	Mono-azo	11270	+	G.T. Gurr	nd	
8. Congo red (CR)	Bis-azo	22120	2−	Merck	nd	
9. Curcumin (Cur)	β-Diketone	75300	0	Merck	+	Bruzell *et al*. 2005
10. Eosin Y (EY)	Hydroxyxanthene	45380	2−	Sigma	+	Seliger & McElroy 1965, Knox & Dodge 1985, Deerinck *et al*. 1994
11. Fast green FCF (FG)	Triphenylmethane	42053	2−	Sigma	nd	
12. Fluorescein (F)	Hydroxyxanthene	45350	2−	Sigma	−	Gandin *et al*. 1983, Devanathan *et al*. 1990
13. Hematoxylin (H)	Neoflavone	75290	−	Panreac	nd	
14. Indigocarmine (IC)	Indigoid	73015	2−	Serva	−	Herkovits *et al*. 2007
15. Janus green B (JGB)	Mono-azo-azine	11050	+	Grübler	nd	
16. Luxol fast blue MBSN ^b^	Phthalocyanine	74180	2−	Merck	− ^d^	Redmond & Gamlin 1999
17. Mercurochrome (Mer)	Hydroxyxanthene	−	2−	Merck	+	Redmond & Gamlin 1999
18. Methylene blue (MB)	Thiazine	52015	+	Sigma	+	Stockert & Herkovits 2003, Smijs *et al*. 2004, Herkovits *et al*. 2007
19. Morin (Mor)	Flavonol	75660	0	Merck	nd	
20. Naphtol Blue Black	Bis-azo	20470	2−	Serva	nd	
21. Neutral red (NR)	Azine	50040	+	Panreac	+	Seliger & McElroy 1965, Barbosa & Peters 1971, Gutter *et al*. 1977
22. NiPcS4 ^c^	Phthalocyanine	−	4−	Aldrich	− ^d^	
23. Nuclear fast red (NFR)	Anthraquinone	60760	−	Merck	+	Kuramoto & Kitao 1981, Robertson *et al*. 2009
24. Phloxine B (PhB)	Hydroxyxanthene	45410	2−	Panreac	+	Rasooly & Weisz 2002, Herkovits *et al*. 2007
25. Primuline (Pri)	Benzothiazole	49000	−	Fluka	nd	
26. Pyronine Y (PY)	Aminoxanthene	45005	+	G.T. Gurr	−	Herkovits *et al*. 2007
27. Rhodamine B (RhB)	Aminoxanthene	45170	+	Merck	+	Ngen *et al*. 2009
28. Riboflavine (Rib)	Alloxazine	−	0	Merck	+	Naseem *et al*. 1988, Redmond & Gamlin 1999
29. Rose bengal (RB)	Hydroxyxanthene	45440	2−	Sigma	+	Banks *et al*. 1985, Marthy *et al*. 1990, Herkovits *et al*. 2007
30. Ruphen ^e^	Ru-diimide	−	2+	Aldrich	−	Dobrucki 2001
31. Safranine O (SO)	Azine	50240	+	Fluka	+	Li *et al*. 2006
32. Tartrazine (Tar)	Mono-azo	19140	3−	Fluka	nd	
33. Thioflavin S (TS)	Benzothiazole	49010	−	Bayer	nd	
34. Thioflavin T (TT)	Benzothiazole	49005	+	G.T. Gurr	+	Seliger & McElroy 1965, Villanueva *et al*. 1993
35. Thionine (T)	Thiazine	52000	+	Merck	nd	
36. Toluidine Blue O (TB)	Thiazine	52040	+	Merck	+	Stockert *et al*. 1996, Herkovits *et al*. 2007, Blázquez-Castro *et al*. 2009
37. Trypan Blue (TryB)	Bis-azo	23850	4-	Fluka	−	Barbosa & Peters 1971, Herkovits *et al*. 2007

(a) The reference list for reported photoactivity is not exhaustive and only reflects a few but relevant citations on the best known photoactive dyes. Reference details are shown as supplementary information (S1 Text). (b) CuPcS2:cooper phthalocyanine disulfonic acid, di-*o*-tolylguanidine salt.(c) Nickel phthalocyanine tetrasulfonic acid, tetrasodium salt. (d) In these compounds, the presence of paramagnetic metals (Cu, Ni) abolishes the photodynamic activity. (e) Ruthenium(II)-tris(phenanthroline) dichloride. nd: non determined

### Statistical analysis

In order to quantify the toxic properties of the compounds in worms, a parameter called *Vitality Rate* (VR) was calculated as the ratio between dye treated worms and contol animals in the same assay. Based on previous experience on tracking *C*. *elegans*, where 10% variations in locomotor activity are common over continous 30 min periods of activity tracking (S1 Table), an activity drop below 0.8 of the control population level (significantly different from 1, p < 0.05 ONE sample t-test) was set to be considered a positive toxic effect.

### Dye uptake

Worms were exposed to dyes as described previously, with the difference that the culture medium with the chemical was removed from the medium after the 1 h incubation, and worms were washed inside microplates 4 times with saline solution (modified K-medium). After this treatment, the dye accumulated inside the animal body was photoactivated for 30 min, and locomotor activity was recorded for 4 hours as described previously. In parallel, 3 replicate wells were observed using bright-field and fluorescence microscopy under blue or green excitation light with the purpose of visually assessing dye uptake. The accumulation of dyes was arbitrarily quantified by the intensity of fluorescence or staining using a 4 color scale.

### Drug response pathway determination

The previously indicated GFP reporter strains were cultured in NGM plates until adulthood, and treated as described above for the behavioral assessment experiments. GFP expression was determined by visual inspection and imaged using a fluorescence stereoscope (OLYMPUS model: SZX-ILLK100) 4 h after dye photoactivation. In addition, RNA samples were prepared from a population of adult worms (SS104 strain) treated with 1 μM rose Bengal, our positive control dye, and *hsp-4* plus *hsp-6* genes were quantified by qRT-PCR using the protocol reported previously by Buzzi *et al* [42]. The *ama-1* gene was employed as a constitutively expressed internal control. Primers used for real time PCR are as follows: for hsp-4, hsp-4F 5’GCAGATGATCAAGCCCAAAAAG3’and hsp4R 5’GCGATTTGAGTTTTCATCTGATAGG3’; for hsp-6, hsp-6F 5’GGACAAACCAAAGGGACATG3’ and hsp-6R 5’AACGAATGCTCCAACCTGAG3’; for ama-1, ama-1F 5’CCTACGATGTATCGAGGCAAA3’ and ama-1R 5’CCTCCCTCCGGTGTAATAATG3’. All experiments were replicated 3 times.

## Results

### Phototoxicity assessment in adult *Caenorhabditis elegans*

**Fig 1:**
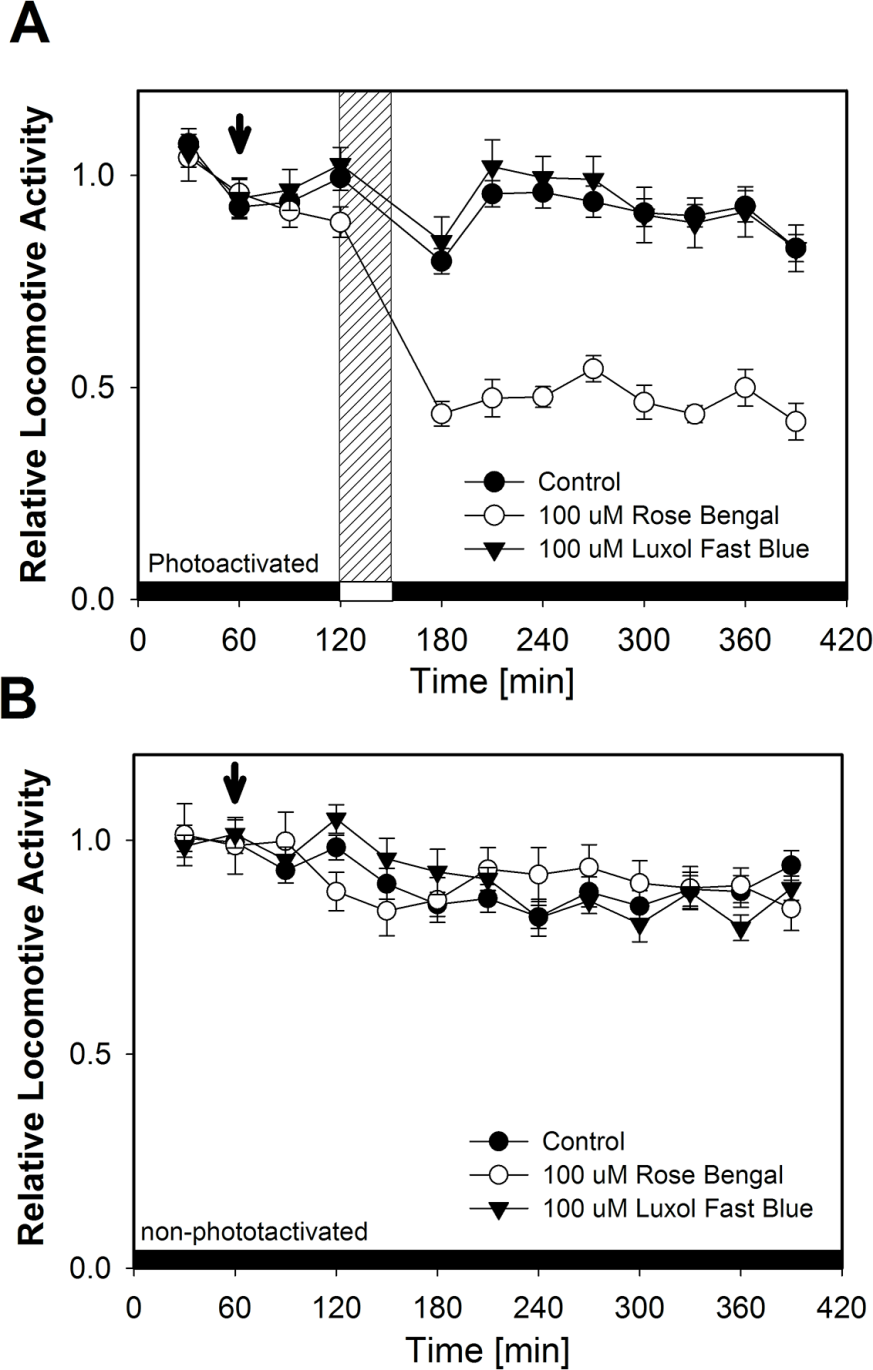
Phototoxic compound activation affects *C*.*elegans* behavior. Worms where incubated in buffer (control), non-phototoxic dye (Luxol Fast Blue) and phototoxic dye (Rose Bengal). Locomotor activity was measured continously using an infrared tracking device (WMicrotracker) with exposure to photoactivating light pulse [30 min white light, 10 mW/cm^2^] (A) or without light exposure (B). Relative Locomotor Activity is reported as the acumulated activity of 30 min time frames over the average activity before adition of compounds.

To perform phototoxicity experiments we developed a stepped protocol, where all animals are subjected to the compounds in the dark and then either exposed to the light (Fig 1A) or kept in darkness as control (Fig 1B). Their motility was recorded with the infrared tracking method for 1 h before exposure to light and 4 h after in order to record the behavioral response. Firstly, two well characterized dyes were tested, Luxol fast blue (not phototoxic) and rose Bengal (a photoactive molecule). Neither caused an effect without light irradiation (Fig 1B).

In contrast, when white light irradiation (10 mW/cm^2^, 30 min) is applied (Fig 1A), a significant reduction in locomotor activity is observed in the positive control (rose Bengal), and it remains low even after 4 h. Unexpectedly, an immediate response is also observed in control animals and with a negative control (Luxol fast blue) just after light irradiation, possibly as a transient response to light. Since this short term response is magnified in animals treated with phototoxic molecules, we decided to divide biological effects in “immediate phototoxicity” and “late phototoxicity”.

**Fig 2:**
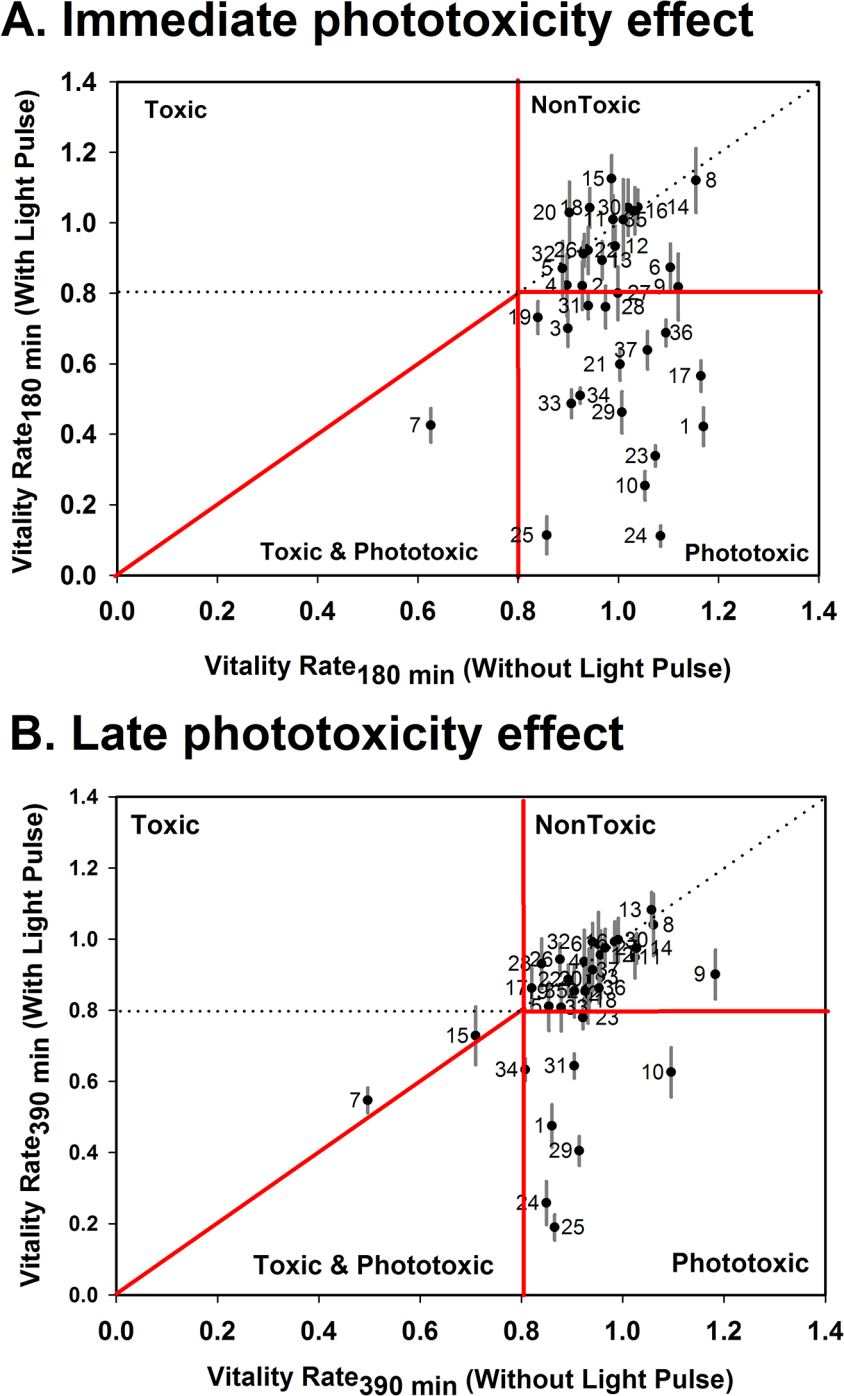
Classification of dye phototoxicity according to behavioral changes. Vitality Rate (VR) index were calculated as [locomotor activity of dye treated worms] / [locomotor activity of control animals]. When observed in a two axis plot (VR_light_ vs. VR_no-light_) toxic effects could be classified as: Toxic (VR_no-light_ < 0.8), Phototoxic (VR_light_ < 0.8) and Toxic and Phototoxic (VR_light_ < VR_no-light_ < 0.8_,_ *p < 0.05). A) Behavioral changes observed 30min after light pulse (180min from start) were classed as “immediate effects”, where 43% of compounds showed phototoxic effects. B) Changes observed 240 min after light pulse (390 min from start) were classed as “late phototoxicity effects”, where 21% of compounds showed phototoxic effects. N = 3 ± SEM.

We then selected a set of 37 dyes to test phototoxic effects in *C*. *elegans* cultures. Of these, 16 have been already reported to present effects in other organisms (Table 1). Of the assessed list of 37 compounds, 16 presented immediate phototoxic effects and 8 late phototoxic effects (Table 2, Fig 2A and 2B). In order of decreasing phototoxicity, the top 5 dyes were phloxine B > primuline > eosin Y > acridine orange > rose Bengal. Many of these dyes have been previously reported as phototoxic, consistent with our results (Table 1).

**Table 2:** Results of the screening for phototoxicity.

		Immediate Phototoxicity (30 min after light pulse)			Late Phototoxicity (240 min after light pulse)		
#	Name	Vitality w/Light Pulse	Vitality non Light Pulse	Classification	Vitality w/Light Pulse	Vitality non Light Pulse	Classification
**24**	**Phloxine B**	0.11 +/- 0.03	1.08 +/- 0.09	**Phototoxic**	0.26 +/- 0.06	0.85 +/- 0.08	**Phototoxic**
**25**	**Primuline**	0.11 +/- 0.05	0.86 +/- 0.07	**Phototoxic**	0.19 +/- 0.04	0.87 +/- 0.07	**Phototoxic**
**10**	**Eosin Y**	0.25 +/- 0.04	1.05 +/- 0.1	**Phototoxic**	0.63 +/- 0.07	1.1 +/- 0.13	**Phototoxic**
**1**	**Acridine orange**	0.42 +/- 0.05	1.17 +/- 0.08	**Phototoxic**	0.48 +/- 0.06	0.86 +/- 0.08	**Phototoxic**
**29**	**Rose bengal**	0.46 +/- 0.06	1.01 +/- 0.07	**Phototoxic**	0.4 +/- 0.04	0.91 +/- 0.05	**Phototoxic**
**34**	**Thioflavin T**	0.51 +/- 0.02	0.92 +/- 0.03	**Phototoxic**	0.63 +/- 0.03	0.81 +/- 0.05	**Phototoxic**
**31**	**Safranine T**	0.76 +/- 0.04	0.94 +/- 0.03	**Phototoxic**	0.64 +/- 0.03	0.9 +/- 0.04	**Phototoxic**
**23**	**Nuclear fast red**	0.34 +/- 0.03	1.07 +/- 0.04	**Phototoxic**	0.78 +/- 0.03	0.92 +/- 0.06	**Phototoxic**
**7**	**Chrysoidine**	0.42 +/- 0.05	0.63 +/- 0.06	**Phot + Tox**	0.55 +/- 0.03	0.5 +/- 0.05	**Toxic**
**33**	**Thioflavin S**	0.49 +/- 0.04	0.91 +/- 0.04	**Phototoxic**	0.81 +/- 0.07	0.88 +/- 0.05	**NonToxic**
**17**	**Mercurochrome**	0.56 +/- 0.04	1.16 +/- 0.06	**Phototoxic**	0.86 +/- 0.06	0.82 +/- 0.05	**NonToxic**
**21**	**Neutral red**	0.6 +/- 0.05	1 +/- 0.05	**Phototoxic**	0.85 +/- 0.04	0.93 +/- 0.04	**NonToxic**
**37**	**Trypan blue**	0.64 +/- 0.05	1.06 +/- 0.05	**Phototoxic**	0.91 +/- 0.06	0.94 +/- 0.09	**NonToxic**
**36**	**Toluidine blue O**	0.69 +/- 0.04	1.09 +/- 0.05	**Phototoxic**	0.86 +/- 0.04	0.95 +/- 0.05	**NonToxic**
**3**	**Auramine O**	0.7 +/- 0.05	0.9 +/- 0.07	**Phototoxic**	0.89 +/- 0.06	0.96 +/- 0.06	**NonToxic**
**28**	**Riboflavine**	0.76 +/- 0.06	0.97 +/- 0.05	**Phototoxic**	0.93 +/- 0.07	0.84 +/- 0.06	**NonToxic**
**27**	**Rhodamine B**	0.8 +/- 0.08	1 +/- 0.05	**Phototoxic**	0.98 +/- 0.05	0.97 +/- 0.04	**NonToxic**
**19**	**Morin**	0.73 +/- 0.05	0.84 +/- 0.08	**Toxic**	0.85 +/- 0.06	0.9 +/- 0.09	**NonToxic**
**9**	**Curcumin**	0.82 +/- 0.09	1.12 +/- 0.09	**NonToxic**	0.9 +/- 0.07	1.18 +/- 0.12	**NonToxic**
**2**	**Alizarin red S**	0.82 +/- 0.07	0.93 +/- 0.08	**NonToxic**	0.85 +/- 0.08	0.93 +/- 0.07	**NonToxic**
**4**	**Berberine**	0.82 +/- 0.09	0.9 +/- 0.05	**NonToxic**	0.94 +/- 0.09	0.92 +/- 0.09	**NonToxic**
**5**	**Bismarck brown Y**	0.87 +/- 0.08	0.89 +/- 0.06	**NonToxic**	0.81 +/- 0.07	0.85 +/- 0.06	**NonToxic**
**6**	**Carmine**	0.87 +/- 0.07	1.1 +/- 0.06	**NonToxic**	0.99 +/- 0.09	0.95 +/- 0.09	**NonToxic**
**13**	**Hematoxylin**	0.89 +/- 0.05	0.97 +/- 0.05	**NonToxic**	1.08 +/- 0.05	1.06 +/- 0.06	**NonToxic**
**32**	**Tartrazine**	0.91 +/- 0.04	0.93 +/- 0.06	**NonToxic**	0.99 +/- 0.05	0.94 +/- 0.05	**NonToxic**
**22**	**NiPcS4**	0.92 +/- 0.07	0.94 +/- 0.05	**NonToxic**	0.89 +/- 0.04	0.89 +/- 0.06	**NonToxic**
**26**	**Pyronine Y**	0.92 +/- 0.05	0.93 +/- 0.03	**NonToxic**	0.94 +/- 0.04	0.88 +/- 0.04	**NonToxic**
**12**	**Fluorescein**	0.93 +/- 0.06	0.99 +/- 0.09	**NonToxic**	0.96 +/- 0.07	0.96 +/- 0.07	**NonToxic**
**35**	**Thionine**	1.01 +/- 0.11	1.01 +/- 0.08	**NonToxic**	0.85 +/- 0.07	0.9 +/- 0.07	**NonToxic**
**11**	**Fast green FCF**	1.01 +/- 0.07	0.99 +/- 0.07	**NonToxic**	0.95 +/- 0.06	1.02 +/- 0.09	**NonToxic**
**20**	**Naphthol Blue Black**	1.03 +/- 0.09	0.9 +/- 0.06	**NonToxic**	0.89 +/- 0.09	0.93 +/- 0.08	**NonToxic**
**16**	**Luxol fast blue**	1.03 +/- 0.07	1.03 +/- 0.06	**NonToxic**	0.99 +/- 0.06	0.98 +/- 0.05	**NonToxic**
**18**	**Methylene blue**	1.04 +/- 0.06	0.94 +/- 0.04	**NonToxic**	0.83 +/- 0.04	0.93 +/- 0.05	**NonToxic**
**30**	**Ruphen**	1.04 +/- 0.08	1.02 +/- 0.07	**NonToxic**	1 +/- 0.06	0.99 +/- 0.08	**NonToxic**
**14**	**Indigocarmine**	1.04 +/- 0.05	1.04 +/- 0.06	**NonToxic**	0.97 +/- 0.04	1.03 +/- 0.1	**NonToxic**
**8**	**Congo red**	1.12 +/- 0.09	1.15 +/- 0.1	**NonToxic**	1.04 +/- 0.09	1.06 +/- 0.11	**NonToxic**
**15**	**Janus green B**	1.12 +/- 0.07	0.99 +/- 0.05	**NonToxic**	0.73 +/- 0.08	0.71 +/- 0.07	**Toxic**

All dyes were tested at least in 2 independent experiments (with 4 internal replicates each) using 100 μM concentration, with the exception of curcumin which was assayed at 10 μM for solubility reasons. Vitality is reported as the rate of locomotor activity of dye treated worms over buffer treated worms under the same light conditions (Vitality Rate).

In order to discard any synthetic effects between SS104 and a particular chemical, we test top 12 dyes in wild type (N2) young adult animals. As shown in S2 Table, although some sensitivity differences are observed, 100% of compounds manifest similar phototoxic behavior response.

### Phototoxic molecules are acting directly from within the animal body

**Fig 3:**
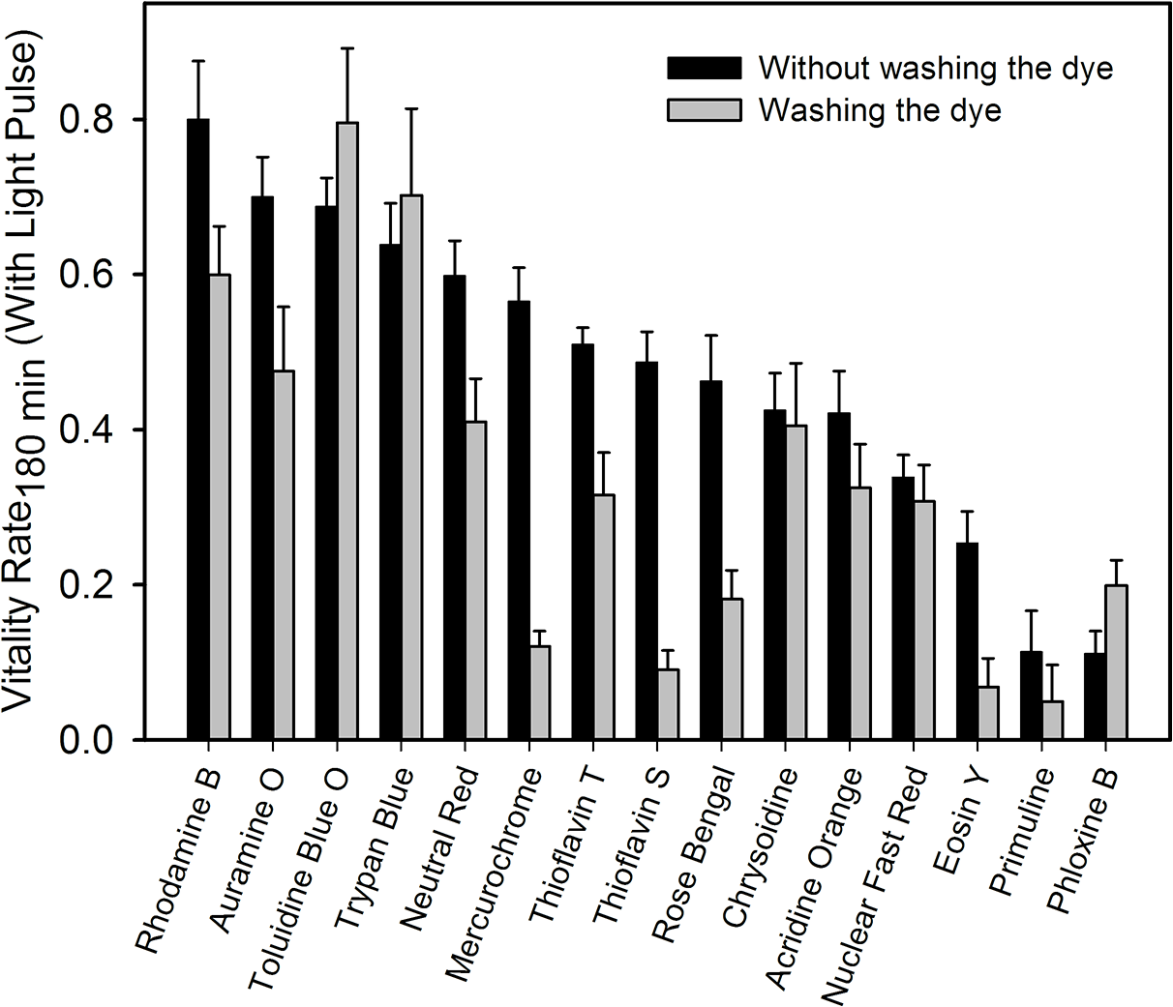
Phototoxic effects remain after drug washing out. The VR of light exposed worms is shown at 180 min (30 min after light pulse) for 15 phototoxic dyes, washing them out of the medium immediately before the light pulse (gray bars) or following the original protocol (black bars). N = 3 ± SEM.

Since we measured phototoxicity without washing the compound from the medium, we asked whether the toxic effects were mainly caused by dye molecules in the culture medium or by those absorbed inside the animal. In order to clarify this point we replicated the experimental setup for 15 positive phototoxic molecules, washing the medium previous to light irradiation. Interestingly, phototoxic activity of all retested compounds remained after washout (Fig 3). Moreover, an increase in the phototoxic effect is observed in most compounds after washout. We hypothesize this increment could be attributed to higher light exposure of the worm, since the dye in the medium can be absorbing some light.

**Fig 4:**
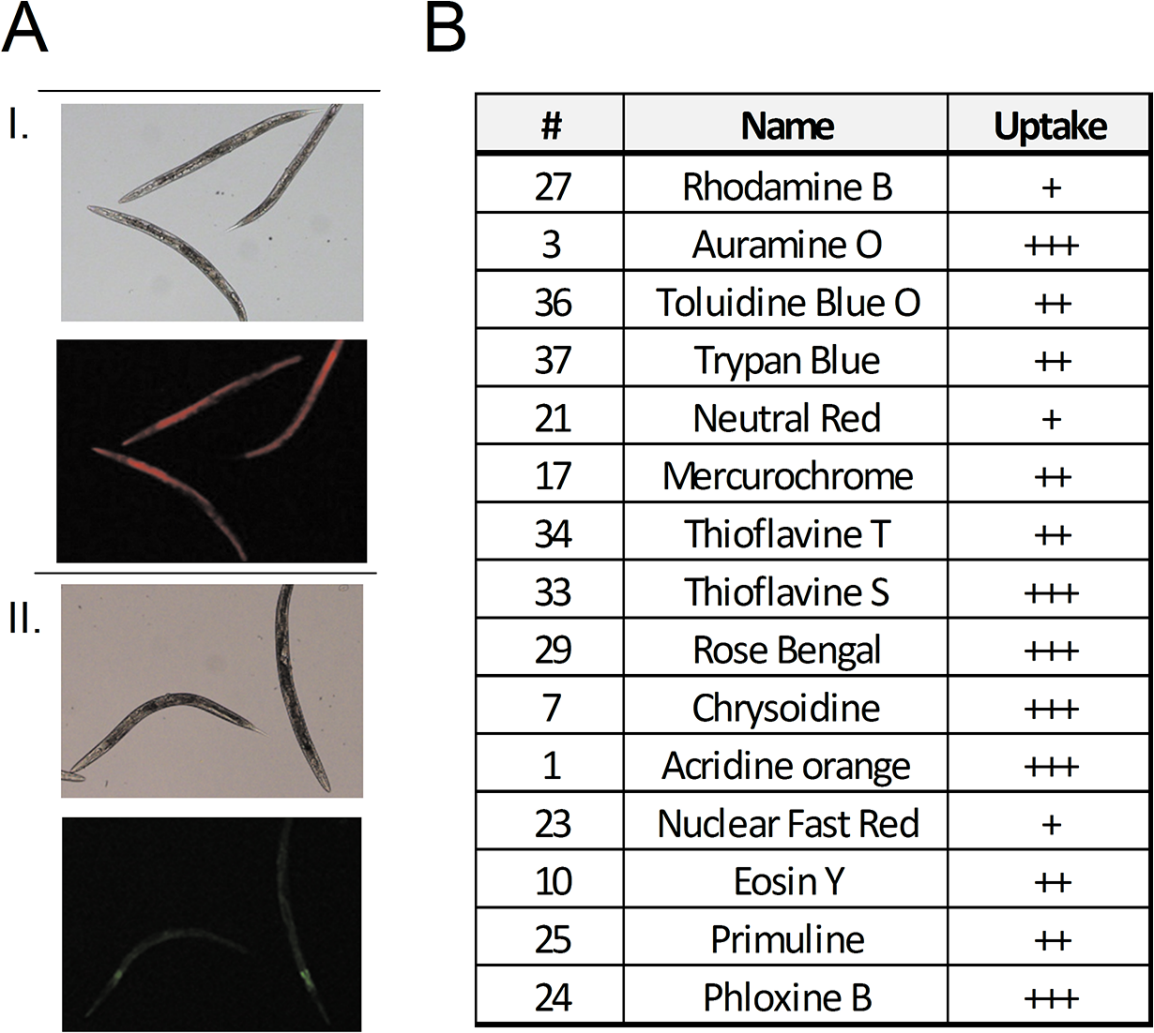
Dye uptake is observed in treated worms. Worm staining or fluorescence (under green or blue light excitation) was visually quantified using a 3 value scale. A) Representative images of worms treated with Rose Bengal (I) under bright-field and green-light excitation, and (II) treatment with thionine observed under bright-field and blue-light excitation. B) Table of dye uptake quantification is shown for 15 phototoxic dyes.

Also, in order to confirm that the compounds did in fact enter the animals, we carried out observations under the stereoscope to assess the staining or fluorescence due to dye uptake. Almost all phototoxic compounds displayed positive staining or fluorescence in living animals, demonstrating the permeability of this animal model to the dyes (Fig 4).

### Stress response genes are turned on in response to some phototoxic molecules in worms

**Fig 5:**
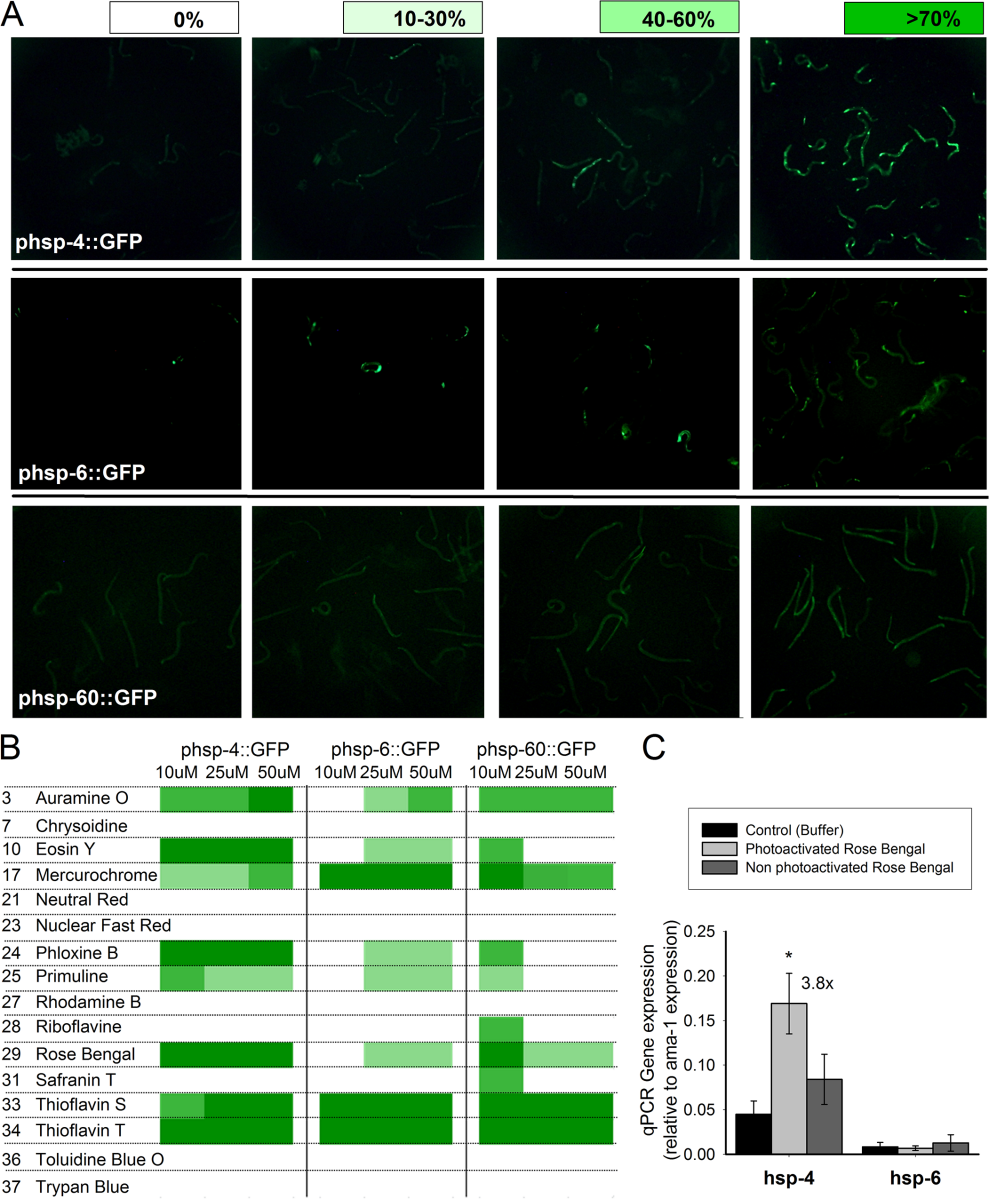
Stress response machinery is activated in *C*. *elegans* under phototoxic treatment. Two mitochondrial-specific Stress Response (SR) lines (hsp-6::GFP and hsp-60::GFP) and one endoplasmic reticulum SR line (hsp-4::GFP) were subjected to the phototoxicity protocol, and GFP-gene expression was observed 4 hours after dye photoactivation. A) Representative scale determination of each reporter line. B) Observed gene expression for 15 phototoxic dyes tested at 10 μM, 25 μM and 50 μM. N = 3. C) Photo-activated Rose Bengal stress response measured by qPCR. Hsp-4 was assayed as a marker of endoplasmic reticulum stress and hsp-6 was assayed as a marker of mitochondrial stress. RNA polymerase subunit gene (ama-1) was employed as internal reference gene. N = 3 ± SEM.

Since it has been reported that phototoxic compounds produce oxidative stress and mitochondrial damage, we studied whether or not the compound were mainly active in any particular cellular compartment. For this purpose two mitochondrial-specific stress response proteins (hsp-6::GFP and hsp-60::GFP) and one endoplasmic reticulum stress response protein (hsp-4::GFP) were used. Of the 16 phototoxic compounds tested (acridine orange was rejected due to its intrinsic green fluorescence), 10 were shown to stress one or both compartments while the other 6 compounds did not seem to be active in perturbing either the mitochondria or the endoplasmic reticulum (Fig 5B).

Finally, to give more support to the idea that our finding on the GFP reporter lines reflected what was happening in the phototoxicity screening, a qRT-PCR was performed on 1 μM rose Bengal exposed glp-4 worms (Fig 5C). At this low concentration, hsp-4 expression was increased 3.8 fold with respect to control (n = 3, p<0.05) while hsp-6 expression remained unaffected. No transcription was found under dark dye treatment (no photoactivation). These results confirm the response pattern observed in the reporter lines.

### Correlation between our screening results and previously reported phototoxicity of compounds

**Fig 6:**
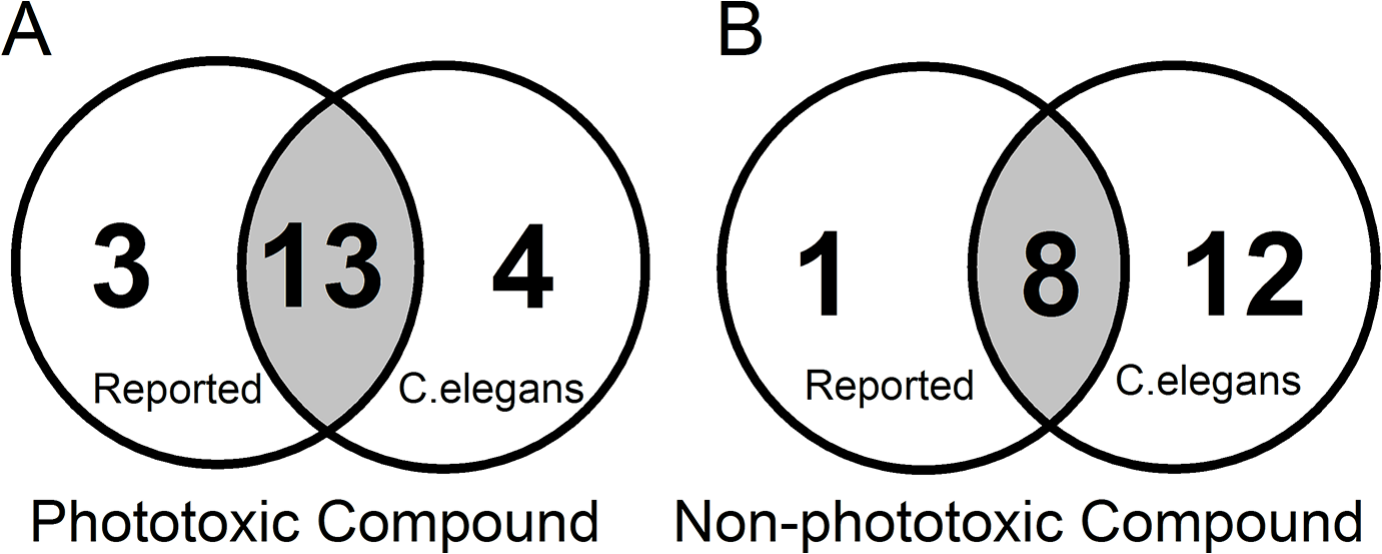
Comparison of worm experiments with previously published data. Results of the current study were compared to reported data for all 37 assayed dyes. Two comparisons are shown: Dyes reported as phototoxic vs. dyes determined as phototoxic in this *C*. *elegans* screening (A) and dyes reported as non-phototoxic vs. dyes determined as non-phototoxic in this *C*. *elegans* screening (B).

A set intersection between our worm experiments and published data is shown in Fig 6. When percentage of false detections is analyzed we found a rate of 8.1% of false negatives (dyes reported as phototoxic, but not detected in our experiments) and 2.7% of false positives (dyes reported as non phototoxic but detected in our system) compared to literature reports. These differences could be attributed to variability in the uptake, efficiency for ROS production, experimental protocol, as well as specific sensitivity and threshold to oxidative stress for distinct type of cells and organisms.

## Discussion

Simple animal models are becoming useful to perform *in vivo* drug discovery experiments and testing for biological activity. In this work we propose the application of the nematode *C*. *elegans* as a reliable model for assessing phototoxicity of dyes as prototypical compounds and the utility of a simple behavioural measurement (such as global locomotor activity) as a direct readout for toxic and sub-toxic effects.

Common photoactivity experiments using cell cultures are based on the incubation with compounds, followed by washing, light exposure and measurement of viability by staining or colorimetric methods. In this work we start the experiments without dye washing. Since similar results were observed after washing of dyes, we conclude that this faster approach can be used in a rapid screening of molecules, previous or complementary to cell culture measurements. At the biological level, photoactive molecules appear to be permeating the animal cuticle or digestive tract, acting from inside the animal body (as shown in uptake experiments, Figs 4 and 5) and resulting in a reduced motility response.

It is worth to note that we were able to measure activation of the stress response machinery in at least 50% of positive compounds (Fig 5). Damage in intracellular compartments associated with protein misfolding and mitochondrial electron transport disruption are well-known mechanisms of phototoxicity action in cell cultures [3]. Our results suggest a similar mechanism of damage in worms. It might be useful to evaluate a larger set of stress-reporter strains and, even more, the amount of ROS driven by dye photoexcitation in *C*. *elegans* in order to gain a deeper understanding of the molecular mechanisms of action. These issues are the objectives of ongoing research.

In order to compare the photodynamic action of each molecule, we can define a Phototoxic Index (PI) by analyzing the phototoxicity and intrinsic toxicity of the compound. The occurrence and degree of photosensitization could be expressed as the ratio: PI = effects with light / effect without light. Thus, the five dyes showing high immediate phototoxicity would present the following values: PI_(phloxine B)_ = 9.81; PI_(primuline)_ = 5.45; PI_(eosin Y)_ = 4.2, PI_(acridine orange)_ = 2.78, and PI_(rose Bengal)_ = 2.19. It should be noted that the higher the PI is, the stronger the phototoxic effect.

Finally, it is important to mention that this animal model and simple protocols for biotoxicity-detection are useful not only for pharmacological testing but also in ecotoxicology assays. Regarding environmental pollution, around 10,000 types of dyes and pigments are produced annually worldwide and extensively used in textile, leather, plastic and printing industries, laboratory work, and as food, pharmaceutical and cosmetic additives. About 10–15% of the total dyes used in dyeing processes are released in wastewater [17]. Therefore, contamination by dyes [19] and the resulting phototoxicity represent a significant risk for human health and wildlife preservation. In addition, other pharmacological agents showing undesired photoactivity must be taken into account (e.g., antiinflammatory, anxiolytic, antirheumatic, antibacterial, and antiparasitic drugs), which have also revealed to be phototoxic [21, 24, 26, 29, 36, 43, 44]. As a logical consequence, it is increasingly necessary to evaluate the phototoxicity of possible drugs or xenobiotics to induce or prevent, respectively, biological effects through the design and development of simple, precise and cheap bioassays for oxidative stress-dependent phototoxicity studies. The present results using an automated tracking device for assessing population motility of a whole organism such as *C*. *elegans* show that this bioassay is very suitable for easy, rapid and accurate evaluation of the phototoxic potential of photosensitizing drugs and environmental pollutants.

## Supporting Information

S1 Table Characterization of activity measurements using wild type animals (N2 strain). In order to estimate the average activity and deviation over a 4 h recording period, 8 biological replicates, independently repeated, are shown in the table. Each test was normalized to the first half hour of recording. Four wells replicates, and 50 worms per well, were used for each experiment.
(DOC)

S2 Table Measurement of response for the top 12 phototoxic compounds using young adults wild type worms (N2 strain). Immediate and late phototoxic response is shown in the table, and compared with SS104 strain results.
(DOC)

S1 Text Supplementary References. (DOCX)
