# Correction: Reliable Screening of Dye Phototoxicity by Using a *Caenorhabditis elegans* Fast Bioassay

**DOI:** 10.1371/journal.pone.0133331

**Published:** 2015-07-15

**Authors:** The PLOS ONE Staff

The third author’s name is spelled incorrectly. The correct name is: Lucila Ines Buzzi. The correct citation is: Bianchi JI, Stockert JC, Buzzi LI, Blázquez-Castro A, Simonetta SH (2015) Reliable Screening of Dye Phototoxicity by Using a *Caenorhabditis elegans* Fast Bioassay. PLoS ONE 10(6): e0128898. doi:10.1371/journal.pone.0128898. The publisher apologizes for this error.

## References

[1] Bianchi JI, Stockert JC, Buzz LI, Blázquez-Castro A, Simonetta SH (2015) Reliable Screening of Dye Phototoxicity by Using a *Caenorhabditis elegans* Fast Bioassay. PLoS ONE 10(6): e0128898 doi: 10.1371/journal.pone.0128898 26039060 10.1371/journal.pone.0128898 PMC4454604

