# Supplementary material for: Reliable Screening of Dye Phototoxicity by Using a Caenorhabditis elegans Fast Bioassay
Source: PLoS One. 2015 Jun 3;10(6):e0128898. doi: 10.1371/journal.pone.0128898 (PMC4454604; doi:10.1371/journal.pone.0128898)
Supplement: S1 Table — In order to estimate the average activity and deviation over a 4 h recording period, 8 biological replicates, independently repeated, are shown in the table. Each test was normalized to the first half hour of recording. Four wells replicates, and 50 worms per well, were used for each experiment. (DOC) [file pone.0128898.s001.doc]

**Supplementary Table 1. Bianchi *et al***

| **Sample / Time (h)** | **0.5** | **1** | **1.5** | **2** | **2.5** | **3** | **3.5** | **4** | **Average**  **(Intra-experiment)** | **Standard deviation** |
| --- | --- | --- | --- | --- | --- | --- | --- | --- | --- | --- |
| **Biological replicate #1** | 100% | 91% | 87% | 91% | 101% | 110% | 101% | 87% | **96%** | **8%** |
| **Biological replicate #2** | 100% | 128% | 117% | 114% | 143% | 104% | 101% | 102% | **113%** | **15%** |
| **Biological replicate #3** | 100% | 116% | 109% | 115% | 95% | 99% | 95% | 87% | **102%** | **10%** |
| **Biological replicate #4** | 100% | 96% | 101% | 97% | 126% | 91% | 103% | 103% | **102%** | **11%** |
| **Biological replicate #5** | 100% | 83% | 81% | 82% | 102% | 87% | 95% | 88% | **90%** | **8%** |
| **Biological replicate #6** | 100% | 96% | 109% | 90% | 88% | 93% | 104% | 95% | **97%** | **7%** |
| **Biological replicate #7** | 100% | 142% | 122% | 106% | 131% | 111% | 121% | 97% | **116%** | **16%** |
| **Biological replicate #8** | 100% | 80% | 80% | 87% | 91% | 108% | 61% | 78% | **86%** | **15%** |
| **Average**  **(Inter-experiment)** | **100%** | **104%** | **101%** | **98%** | **110%** | **100%** | **98%** | **92%** |  |  |
| **Standard deviation** | **0%** | **22%** | **16%** | **13%** | **21%** | **9%** | **17%** | **8%** |  |  |
