# Supplementary material for: Reliable Screening of Dye Phototoxicity by Using a Caenorhabditis elegans Fast Bioassay
Source: PLoS One. 2015 Jun 3;10(6):e0128898. doi: 10.1371/journal.pone.0128898 (PMC4454604; doi:10.1371/journal.pone.0128898)
Supplement: S2 Table — Immediate and late phototoxic response is shown in the table, and compared with SS104 strain results. (DOC) [file pone.0128898.s002.doc]

***Supplementary Table 2. Bianchi et al***

|  | | **Immediate Phototoxicity (30 min after light pulse)** | | | | **Late Phototoxicity (240 min after light pulse)** | | | |
| --- | --- | --- | --- | --- | --- | --- | --- | --- | --- |
|  |  | **Effect on N2 strain** | | | **Effect on SS104 strain** | **Effect on N2 strain** | | | **Effect on SS104 strain** |
| **#** | **Name** | **Vitality w/Light Pulse** | **Vitality non Light Pulse** | **Classif.** | **Classif.** | **Vitality w/Light Pulse** | **Vitality non Light Pulse** | **Classif.** | **Classif.** |
| **24** | **Phloxine B** | 0.09 +/- 0.05 | 0.82 +/- 0.06 | **Phototoxic** | **Phototoxic** | 0.09 +/- 0.05 | 0.76 +/- 0.04 | **Phototoxic** | **Phototoxic** |
| **25** | **Primuline** | 0 +/- 0 | 0.82 +/- 0.13 | **Phototoxic** | **Phototoxic** | 0.01 +/- 0.01 | 0.87 +/- 0.05 | **Phototoxic** | **Phototoxic** |
| **10** | **Eosin Y** | 0.02 +/- 0.02 | 1.05 +/- 0.1 | **Phototoxic** | **Phototoxic** | 0.14 +/- 0.07 | 1.11 +/- 0.03 | **Phototoxic** | **Phototoxic** |
| **1** | **Acridine orange** | 0.17 +/- 0.03 | 1.04 +/- 0.03 | **Phototoxic** | **Phototoxic** | 0.56 +/- 0.11 | 0.76 +/- 0.13 | **Phototoxic** | **Phototoxic** |
| **29** | **Rose bengal** | 0.01 +/- 0.01 | 0.53 +/- 0 | **Phot + tox** | **Phototoxic** | 0 +/- 0 | 0.55 +/- 0.01 | **Phot + tox** | **Phototoxic** |
| **34** | **Thioflavin T** | 0.31 +/- 0.15 | 1.02 +/- 0.2 | **Phototoxic** | **Phototoxic** | 0.14 +/- 0.07 | 0.87 +/- 0.24 | **Phototoxic** | **Phototoxic** |
| **31** | **Safranine T** | 0.17 +/- 0.1 | 0.67 +/- 0.2 | **Phototoxic** | **Phototoxic** | 0.08 +/- 0.06 | 0.58 +/- 0.07 | **Phot + tox** | **Phototoxic** |
| **23** | **Nuclear fast red** | 0.53 +/- 0.01 | 0.74 +/- 0.07 | **Phototoxic** | **Phototoxic** | 0.75 +/- 0.06 | 0.8 +/- 0.07 | **NonToxic** | **Phototoxic** |
| **7** | **Chrysoidine** | 0.65 +/- 0.32 | 0.89 +/- 0 | **Phototoxic** | **Phot + Tox** | 0.73 +/- 0.37 | 0.73 +/- 0 | **Toxic** | **Toxic** |
| **33** | **Thioflavin S** | 0.12 +/- 0.07 | 1.11 +/- 0.21 | **Phototoxic** | **Phototoxic** | 0.35 +/- 0.16 | 0.9 +/- 0.01 | **Phototoxic** | **NonToxic** |
| **17** | **Mercurochrome** | 0.03 +/- 0.01 | 1.2 +/- 0.17 | **Phototoxic** | **Phototoxic** | 0.33 +/- 0.18 | 0.69 +/- 0.04 | **Phot + tox** | **NonToxic** |
| **21** | **Neutral red** | 0.04 +/- 0.02 | 0.88 +/- 0.02 | **Phototoxic** | **Phototoxic** | 0.11 +/- 0.03 | 0.75 +/- 0.02 | **Phototoxic** | **NonToxic** |
