# Supplementary material for: Reliable Screening of Dye Phototoxicity by Using a Caenorhabditis elegans Fast Bioassay
Source: PLoS One. 2015 Jun 3;10(6):e0128898. doi: 10.1371/journal.pone.0128898 (PMC4454604; doi:10.1371/journal.pone.0128898)
Supplement: S1 Text — (DOCX) [file pone.0128898.s003.docx]

**S1 Text Reference List:**

Alvarez M, Villanueva A, Acedo P, Cañete M, Stockert JC (2011) Cell death causes relocalization of photosensitizing fluorescent probes. Acta Histochem 113: 363-368.

Banks JG, Board RG, Carter J, Dodge AD (1985) The cytotoxic and photodynamic inactivation of micro-organisms by Rose Bengal. J Appl Bacteriol 58: 391-400.

Barbosa P, Peters TM (1971) The effects of vital dyes on living organisms with special reference to Methylene Blue and Neutral Red. Histochem J 3: 71-93.

Blázquez-Castro A, Stockert JC, Sanz-Rodríguez F, Zamarrón A, Juarranz A (2009) Differential photodynamic response of cultured cells to methylene blue and toluidine blue: role of dark redox processes. Photochem Photobiol Sci 8: 371-376.

Bruzell EM, Morisbak E, Tønnesen HH (2005) Studies on curcumin and curcuminoids. XXIX. Photoinduced cytotoxicity of curcumin in selected aqueous preparations. Photochem Photobiol Sci 4: 523-530.

Deerinck TJ, Martone ME, Lev.Raam V, Green DPL, Tsien RY, Spector DL, Huang S, Ellisman MH (1994) Fluorescence photooxidation with eosin: A method for high resolution immunolocalization and in situ hybridization detection for light and electron microscopy. J Cell Biol 126: 901-910.

Devanathan S, Dahl TA, Midden WR, Neckers DC (1990) Readily available fluorescein isothiocyanate-conjugated antibodies can be easily converted into targeted phototoxic agents for antibacterial, antiviral, and anticancer therapy. Proc Nat Acad Sci USA 87: 2980-2984.

Dobrucki JW (2001) Interaction of oxygen-sensitive luminescent probes Ru(phen)^2+^_3_ and Ru(bipy)^2+^_3_ with animal and plant cells in vitro. Mechanism of phototoxicity and conditions for non-invasive oxygen measurements. J Photochem Photobiol B: Biol 65: 136-144.

Gandin E, Lion Y, Van de Vorst A (1983) Quantum yield of singlet oxygen production by xanthene derivatives. Photochem Photobiol 37: 271-278.

Gutter B, Speck WT, Rosenkranz HS (1977) Light-induced mutagenicity of neutral red (3-amino-7-dimethylamino-2-methylphenazine hydrochloride).Cancer Res 37: 1112-1114.

Herkovits J, Pérez-Coll CS, Stockert JC, Blázquez A (2007) The screening of photodynamic toxicity of dyes by means of a bioassay using amphibian embryos. Res J Chem Environ 11: 86-91.

Inbaraj JJ, Kukielczak BM, Bilski P, Sandvik SL, Chignell CF (2001) Photochemistry and photocytotoxicity of alkaloids from Goldenseal (*Hydrastis canadensis* L.) 1. Berberine. Chem Res Toxicol 14: 1529-1534.

Knox JP, Dodge AD (1985) The photodynamic action of eosin, a singlet oxygen generator. The inhibition of photosynthetic electron transport. Planta 164: 30-34.

Kuramoto N, Kitao T (1981) Mechanism of the photofading of dye: contribution of singlet oxygen to the photofading of aminoanthraquinone. Dyes & Pigments 2: 133-141.

Li YS, Wang ZT, Luo SD, Li SS, Zhu DY (2006) Sensitized photooxidation of furanoeremophilane with singlet oxygen and their biogenetic relationship. Nat Prod Res 20: 724-730.

Marthy HJ, Murasecco-Suardi P, Oliveros E, Braun AM (1990) Primary effects of singlet oxygen sensitizers on eggs and embryos of sea urchin. J Photochem Photobiol B: Biol 7: 303-315.

Molero ML, Hazen MJ, Stockert JC (1985) Photodynamic effect of berberine sulfate on the growth rate of *Allium cepa* roots. J Plant Physiol 120: 91‑94.

Naseem I, Ahmad M, Hadi SM (1988) Effect of alkylated and intercalated DNA on the generation of superoxide anion by riboflavin. Bioscience Rep 8: 485-492.

Ngen EJ, Rajaputra P, You Y (2009) Evaluation of delocalized lipophilic cationic dyes as delivery vehicles for photosensitizers to mitochondria. Bioorg Med Chem 17: 6631-6640.

Rasooly A, Weisz A (2002) In vitro antibacterial activities of phloxin B and other halogenated fluoresceins against methicillin-resistant *Staphylococcus aureus*. Antimicrob Agents Chemother 46: 3650-3653.

Redmond RW, Gamlin JN (1999) A compilation of singlet oxygen yields from biologically relevant molecules. Photochem Photobiol 70: 391-475.

Robertson PKJ, Black KD, Adams M, Willis K, Buchan F, Orr H, Lawton L, McCullach C (2009) A new generation of biocides for control of crustacean in fish farms. J Photochem Photobiol B: Biol 95: 58-63.

Seliger HH, McElroy WD (1965) Light: physical and biological action. Acad Press: New York, London. pp 324-332.

Smijs TGM, Nivard MJM, Schuitmaker HJ (2004) Development of a test system for mutagenicity of photosensitizers using *Drosophila melanogaster*. Photochem Photobiol 79: 332-338.

Stockert JC, Del Castillo P, Llorente AR, Rasskin DM, Romero JB, Gómez A (1990) New fluorescence reactions in DNA cytochemistry. 1. Microscopic and spectroscopic studies on nonrigid fluorochromes. Analyt Quant Cytol Histol 12: 1-10.

Stockert JC, Juarranz A, Villanueva A, Cañete M (1996) Photodynamic damage to HeLa cell microtubules induced by thiazine dyes. Cancer Chemother Pharmacol 39: 167-169.

Stockert JC, Herkovits J (2003) Photodynamic toxicity and its prevention by antioxidative agents in *Bufo arenarum* embryos. Toxicology 192: 211-218.

Villanueva A, Cañete M, Trigueros C, Rodríguez-Borlado L, Juarranz A (1993) Photodynamic induction of DNA-protein cross-linking in solution by several photosensitizers and visible light. Biopolymers 33: 239-244.

Zdolsek JM, Olsson GM, Brunck UT (1990) Photooxidative damage to lysosomes of cultured macrophages by acridine orange. Photochem Photobiol 51: 67-76.
